# Antioxidant and Hepatoprotective Effects of *Moringa oleifera*-mediated Selenium Nanoparticles in Diabetic Rats.

**DOI:** 10.12688/f1000research.159362.1

**Published:** 2025-01-02

**Authors:** Anas Ahzaruddin Ahmad Tarmizi, Nik Nasihah Nik Ramli, Maisarah Abdul Mutalib, Nor Amira Jasmi, Mohd Helmy Mokhtar, Siti Hajar Adam

**Affiliations:** 1 School of Graduate Studies, Management and Science University, Shah Alam, Selangor, 40100, Malaysia; 2 Department of Physiology, Faculty of Medicine, Universiti Kebangsaan Malaysia, Kuala Lumpur, Wilayah Persekutuan Kuala Lumpur, 5600, Malaysia; 3 Pre-Clinical Department, Faculty of Medicine and Defence Health, Universiti Pertahanan Nasional Malaysia, Kuala Lumpur, Federal Territory of Kuala Lumpur, 5700, Malaysia

**Keywords:** Moringa oleifera, selenium nanoparticles, diabetes mellitus, antioxidant, hepatoprotective, eco-friendly synthesis, public health.

## Abstract

**Background:**

The search for efficient treatments for type 2 diabetes mellitus (T2DM) has highlighted the potential of plant-based therapeutic compounds and eco-friendly processes for producing selenium nanoparticles. This study investigates the antidiabetic potential of *Moringa oleifera*-mediated biogenic selenium nanoparticles (MO-SeNPs) in diabetic rats.

**Methods:**

Male *Sprague-Dawley* rats were induced with diabetes via a high-fat diet for 2 weeks followed by a single intraperitoneal injection of streptozotocin (STZ) at 45 mg/kg body weight (BW). The rats were divided into five groups: normal, diabetic control, metformin at 100 mg/kg/BW, and two groups treated with oral administration of MO-SeNPs at 0.25 and 0.5 mg/kg body weight for 28 days. Food and water intake as well as fasting blood glucose and body weight were measured weekly. After the treatment period, rats were sacrificed, and blood and liver samples were harvested for further analysis.

**Results:**

MO-SeNPs treatment significantly reduced blood glucose levels ( *p* < 0.05) and restored insulin resistance, with lower dose demonstrating better glycaemic control than larger dose. MO-SeNPs also increased hepatic antioxidant enzyme activity, including GSH-Px, CAT, and T-SOD, which neutralise oxidative stress. MO-SeNPs also improves cardiovascular health by raising HDL and lowering LDL. MO-SeNPs showed hepatoprotective benefits by lowering inflammatory markers such TNF-α, IL-6, IL-1β, iNOS, and AGEs, and reduced lipid peroxidation. Diabetes raises inflammatory indicators, causing liver damage and other problems. The reduction in these indicators shows MO-SeNPs reduce liver inflammation and protect the liver. The normalisation of liver enzyme levels (ALT, AST, ALP) showed improved liver function.

**Conclusions:**

The findings suggest that the green synthesis of SeNPs using *Moringa oleifera* offers a viable alternative for diabetes treatment, highlighting its potential to enhance glycemic control and improve overall metabolic health.

## Introduction

Diabetes mellitus is a condition that can be described as a metabolic disorder that cause by disturbed insulin function. ^1^ It is a debilitating syndrome that impede with many biological systems in the body since it involves imbalance homeostasis of the body. ^2^ Oxidative stress is one of the major pathways that can cause complications and worsening of diabetes mellitus. As this event progressed, the degree of oxidative radical produced outnumbered the level of the defence system by the antioxidant, resulting in harmful effects on the body. ^3^ Hyperglycaemic conditions may also aggravate the oxidative stress due to increased reactive oxidative species (ROS). Besides, these ROS impeded with insulin signalling by the phosphatidylinositol 3-kinase (PI3K/Akt) pathway, hence causing pancreatic β-cell disorder, and triggers the inflammatory pathways. ^4^ Ultimately, both oxidative stress and inflammatory reaction contributes in exaggeration of diabetes complication, causing damage to multiple organs including liver. ^5^ This relationship between hyperglycaemia and increased oxidative stress would repeat it vicious cycle if there is no intervention on both conditions. ^6^ Urbanisation that happens through the decades lead towards sedentary-prone lifestyle causing more people to become obese are deemed to be one of the main factors for increase in type 2 diabetes mellitus (T2DM). ^7^ The steady increasing pattern in the T2DM case in Malaysia, alongside its complication requires a major concern from the public health sector. Thus, this incident has led researcher in finding new treatment as therapeutic mediator from a natural resource for more effective utilization for diabetes management and treatment.

Selenium, a natural metalloid element, is an essential micronutrient in humans, plants and animals. ^8^ Dietary selenium can be obtained through daily nourishment, and its inorganic form were absorbed and transported via a sodium-facilitated system at the intestinal membrane to the liver for protein synthesis. ^9^ Amino acid containing selenium known as selenocysteine, acted as the vital component of selenoprotein, which is essential in maintaining redox homeostasis, inflammatory response regulation, immune system modulation and reproduction. ^10, 11^ Despite its significant pharmacological potential, selenium’s narrow therapeutic window poses a risk of toxicity, thus limiting its functional utilization. In recent years, selenium nanoparticles (SeNPs) have emerged as a superior alternative due to their enhanced bioavailability, stability and lower toxicity. ^8, 12^ Furthermore, SeNPs reported to exhibit various therapeutic and medical benefits, including antidiabetic, antioxidant, antimicrobial, and anticancer effects. ^13^

*Moringa oleifera* have been identified to contain various active constituents such as phenolic compound, alkaloid, carotenoid and terpenoid, which believe to be vital for acting as the reducing and capping agent for the green synthesis of SeNPs. ^14^ Besides that, the utilisation of *M. oleifera* as the main plant option was due to its great cultivation rate in this country, making it to be readily available easier to be utilized. ^15^ Green synthesis using plant resources have become more popular as it claimed to be environmentally and budget friendly, compared to the use synthetic and chemical resource. ^16^ Hence, in this project, we aim to investigate the antioxidant and hepatoprotective effects of phytofabricated SeNPs synthesised with *M. oleifera* towards diabetic rats as a possible alternative for T2DM management.

## Methods

### Materials

Streptozotocin (Cat #HY-13753) were purchased from MedChemExpress, Monmouth, NJ, USA. Metformin tablet (500 mg) was purchased from BIG Pharmacy, Malaysia. Insulin (Cat #E-EL-R3034), GSH-Px (Cat #E-BC-K096-M), CAT (Cat #E-BC-K031-M), T-SOD (Cat #E-BC-K020-M), MDA (Cat #E-BC-K025-M), IL-6 (Cat #E-EL-R0015), TNF-α (Cat #E-EL-R2856), ΙL-1β (Cat #E-EL-R0012), iNOS (Cat #E-EL-R0520) and BCA Protein (Cat #E-BC-K318-M) ELISA assay kit were purchased from Elabscience, Houston, Texas USA. AGE (Cat #CSB-E09413r) ELISA assay kit was obtained from CusaBio, Houston, TX, USA.

### *Moringa oleifera*-mediated selenium nanoparticles (MO-SeNPs) preparation

The chemicals used in this study included sodium selenite (Na _2_SeO _3_, ≥98%, CAS No. 10102-18-8), 2,2-diphenyl-1-picrylhydrazyl (DPPH, CAS No. 1898-66-4), sodium hydroxide (NaOH, CAS No. 1310-73-2), hydrochloric acid (HCl, CAS No. 7647-01-0), potassium ferricyanide (K _3_[Fe (CN) _6_], CAS No. 13746-66-2), ferric chloride (FeCl _3_, CAS No. 7705-08-0), trichloroacetic acid (C _2_HCl _3_O _2,_ CAS No. 76-03-9), phosphate buffer solution (PBS, P4417) and ascorbic acid (C _6_H _8_O _6_, CAS No. 50-81-7). All the chemicals are analytically graded and sourced from Merck (West Point, PA). Fresh *Moringa oleifera* leaves were collected from a local supplier in Sungai Petani, Kedah, Malaysia, and identified by a botanist from Universiti Putra Malaysia (Voucher No. MFI 0213/21). The leaves were thoroughly washed, oven-dried at 40 °C for 72 hours, and ground into a fine powder. For the aqueous extract, 20 g of powdered leaves were homogenized in 800 mL of boiling distilled water, shaken at 150 rpm for 4 hours, centrifuged at 4000 rpm for 20 minutes, and filtered using Whatman filter paper No. 1 (Cat No. 1001 125) from GE Healthcare (New Jersey, USA). Selenium nanoparticles (MO-SeNPs) were synthesized by adding 5 mL of a 50 mM sodium selenite solution dropwise to 20 mL of *Moringa oleifera* extract under magnetic stirring, followed by incubation at 37 °C for 48 hours at pH 8 to facilitate the green synthesis. All of these green synthesis of MO-SeNPs was adapted from our previous work. ^17^ The leaves extract of *M. oleifera* was employed to reduce 50 mM sodium selenite solution under magnetic stirring condition. Optimisation of the synthesis led to production of red amorphous selenium nanoparticle deposits and the suspension were collected for cleaning and characterization. MO-SeNPs imaging showed that it has a spherical structure and have the mean sizes of 95.36 nm.

### Experimental animals

Since animals may experience pain, researchers must evaluate the expected consequences on laboratory animals to minimise injury. Researchers must minimise animal pain and foster well-being. Suffering encompasses pain, hunger, thirst, starvation, extreme temperatures, anxiety, stress, injuries, infections, and inability to behave normally. Trapping, labelling, anaesthetising, breeding, transporting, and stabilising might cause suffering before and after the experiment, therefore researchers must examine all of it. Researchers must also consider pre- and post-experiment adaption periods.

Hence, in this study, the *in vivo* rat study was conducted on male Sprague-Dawley rats in Animal House, Science Lab, Level 9, located in Management and Science University (MSU) after ethic approval for animal experiment from MSU Ethic Committee (EA-L3-01-SGS-2024-03-0002). A total of 30 male rats, with the weight ranged around 180-220 g was purchased and stationed in Animal House at MSU. The animals were placed in common conditions of the animal house in polypropylene cages, supplied with water and food *ad libitum.* The rats were kept in a conducive environment with 12:12 h light and dark cycle with air-conditioned at 21-24°C to maintain the relative humidity between 30–70% throughout the experimental process. All the rats were admitted with continuous access of food and water without showing distress such as diarrhea, restlessness or dulling of fur during the observational period. After a period of adaptation (± 72 hours), the animals were randomly divided into five groups. Induction of T2DM was done through high-fat diet (HFD) with Streptozotocin (STZ) injection following the method of previous study with slight modification, ^18^ whereby the rats were randomly selected and normal control group was fed with common chow diet (standard) while the rest of the diabetic groups were placed on a HFD for inducing prediabetes condition for two weeks period as desired. The HFD was prepared using a recipe formulation from previous study where it was made using 50% commercial chow pellet, 24% ghee, 20% full cream milk powder and 6% corn starch flour. ^19^ After two weeks, single intraperitoneal injection of low dose freshly prepared STZ (45 mg/kg body weight, BW) diluted in 0.1M sodium citrate buffer (pH 4.5) were administered to the overnight fasted rats from the diabetic group. Fasting blood glucose level (FBG) was observed with a blood glucometer device (On Call Plus, ACON, USA) by tail-pricked method after a week of induction with STZ. Rats with FBG level more than 16.7 mmol/L were considered as diabetic and selected for the study. ^20^ The rats selected as the normal control were administered with a vehicle citrate buffer (0.25 ml/kg BW) at the same time. All the rats were fed with a normal chow diet after the induction of STZ. The FBG level and body weights of the rats were observed weekly, before and after injection of STZ while food and water intake were measured post STZ administration.

### Experimental design

After the confirmation of T2DM induction, the experimental rats were grouped as follows (n=6): Group 1: Normal control (NC) rats with vehicle (sodium carboxymethylcellulose, Na-CMC)

Group 2: Diabetic control (DC) rats with vehicle

Group 3: Positive control (PC) diabetic rats treated with 100 mg/kg BW of MET

Group 4: Diabetic rats treated with 0.25 mg/kg BW of MO-SeNPs (Tx 0.25)

Group 5: Diabetic rats treated with 0.5 mg/kg BW of MO-SeNPs (Tx 0.5)

The selection of doses for selenium nanoparticle and metformin (MET) were according to previous study. High dosage of 4 mg/kg BW for selenium nanoparticles have been reported to produce therapeutic effect. However, high doses of selenium nanoparticle have been associated with insulin resistance. ^21^ Thus, low and medium doses of selenium nanoparticle were chosen for this study with comparison to standard antidiabetic drug. After of four weeks of daily oral administration of treatment finished, all the rats were weighed, anesthetized with a mixture of ketamine and xylazine and the blood was extracted from the abdominal aorta via cardiac puncture and was collected into blood collection vacutainer tube. Serum was isolated from blood sample by centrifuging at 4000 rpm for 15 min. The liver tissues were excised and weighed, washed with phosphate buffer solution (pH 7.4, 4 °C) and blood serum and liver tissue were kept at -80 ° C for further analyses.

### Assessment of insulin resistance and β-cell function

Homoeostasis model assessment (HOMA) of β-cell function (HOMA-β%) and insulin resistance (HOMA-IR) was performed using fasting serum insulin (μIU/mL) and fasting blood glucose (mg/dL) using the following formula: IR (HOMA-IR) = [fasting glucose (mg/dl) × fasting insulin (μIU/ml)]/405. ^22^ For β-cell function, it will be calculated according to this formula: (HOMA-β) % = [360 × fasting insulin (μIU/mL)]/(fasting glucose (mg/dL)– 63). ^23^ The measurement of insulin level was done using ELISA kit according to the manufacturer’s instructions.

### Serum biochemical analysis

The liver function was studied by measuring liver enzymes such as ALT, AST, ALP while lipid profile was analysed by observing the level of lipid biomarkers such as LDL, HDL, cholesterol and TGL. The biochemical serum biomarkers were analysed based on standard method using Biosystem BA400 chemistry analyser (Biosystem SA, Barcelona, Spain).

### Assessment of antioxidant enzyme and inflammatory biomarker status

The serum samples and liver tissue were subjected to analysis of antioxidant enzyme activities and inflammatory cytokine level. The samples and supernatants were prepared according to the instruction from the manufacturer of ELISA kits. Serum antioxidants enzyme activities were evaluated by testing the glutathione peroxidase (GSH-Px), catalase (CAT), total superoxide dismutase (T-SOD) and whereas the lipid peroxidation in the liver were determined by malondialdehyde (MDA) level using hepatic tissue sample. Analysis of pro-inflammatory cytokine levels were done towards AGE, IL-6, IL-1β, iNOS and TGF-α using the hepatic tissue sample.

### Statistical analysis

Statistical analysis of the results was done using GraphPad Prism 10 software (version 10.2.1)(RRID:SCR_002798)(GraphPad Software Inc., San Diego, CA, USA). All the data acquired throughout the experimental period were recorded in Microsoft Excel. Recorded body weight, biochemical parameter analysis, antioxidant enzyme assays, inflammatory biomarkers analysis, were conducted with analysis of variance (ANOVA) with Tukey’s post hoc test to compare the differences among the treatment groups. The differences were considered as statistically significant when *p-value < 0.05.*

## Results

### Food and water intake changes

**Figure 1.**
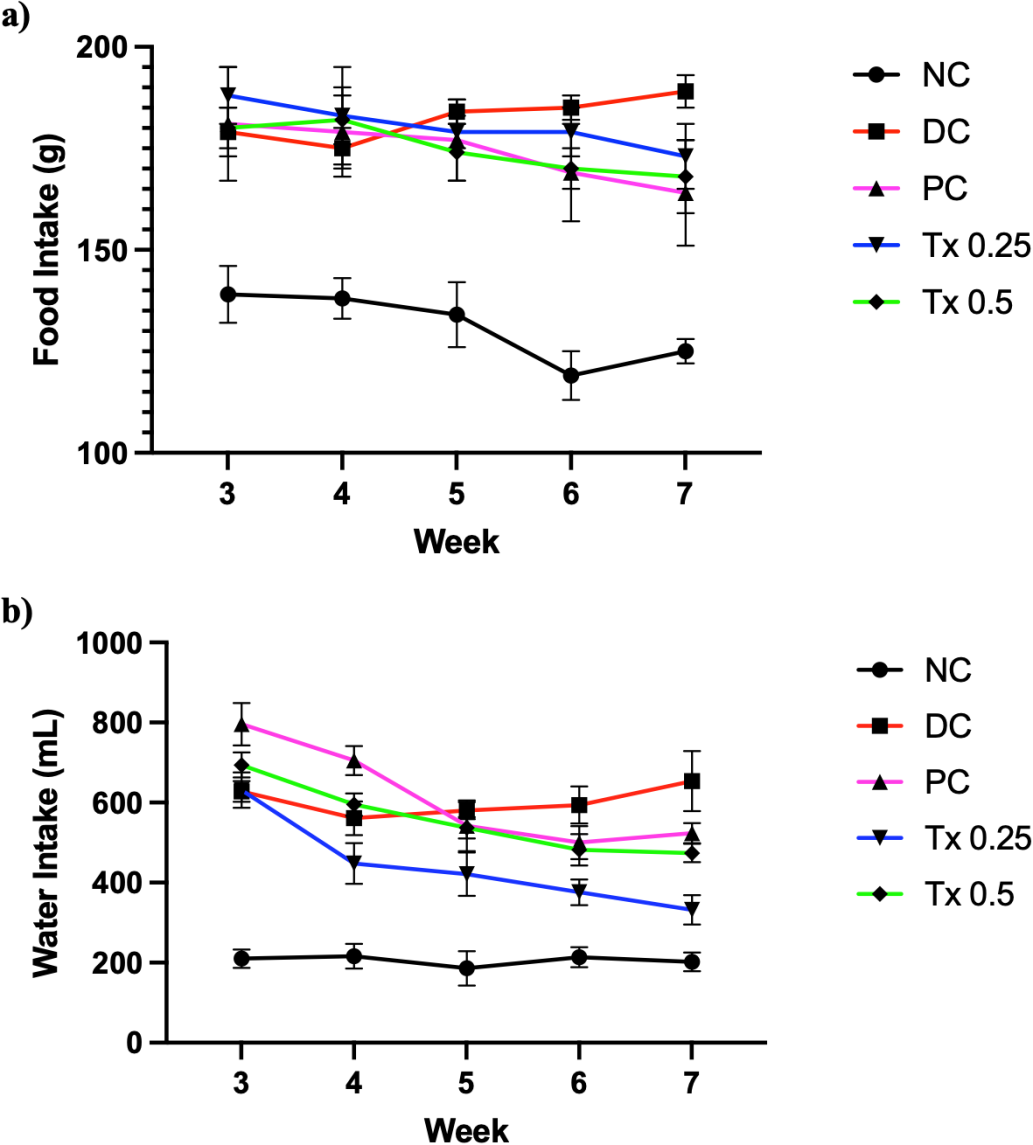
Changes in food (a) and water (b) intake pattern of all experiment groups during four weeks of treatment. Graph points are expressed as mean with SEM as error bar (n=6).

Based on Figure 1, the consumption of normal chow diet and water intake after HFD/STZ induction was measured and observed to be distinct between each group even though the animal groups received same amount of food and water source every day. From Figure 1a, animal groups that received HFD/STZ generally consumed almost 2-fold of the amount from the NC group initially. With treatments commencement, their food intake gradually decreases in level while DC group showed steady increase in food intake. Similar pattern can be seen for the animals’ water intake based from Figure 1b, where at first the rats with diabetic induction showed higher intake of water at more than 3 fold from NC group. Throughout the treatment, treated rats showed reduction in water intake while the DC group maintain its increase until the end of experiment.

### Body weight analysis

**Figure 2.**
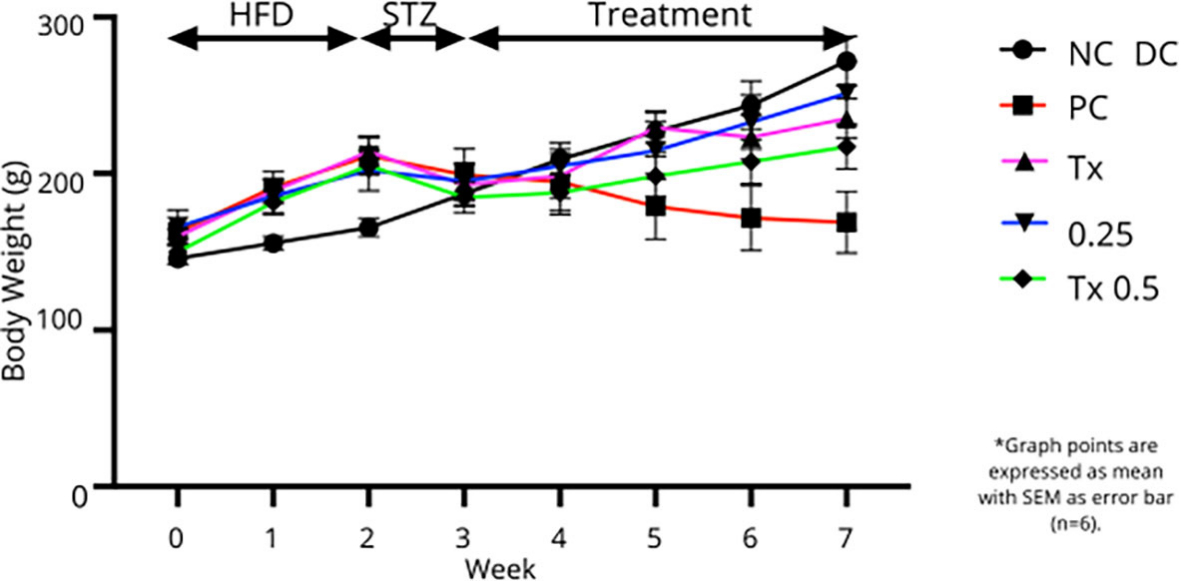
Body weight changes of the treatment groups during the experimental stage. Graph points are expressed as mean with SEM as error bar (n=6).

Figure 2 showed that animal groups that received HFD at the first two weeks of experimental period gained substantial body weight as compared to NC rats that consumed only normal chow diet. After a week of STZ injection, all the subjected rats showed a reduction in body weight. However, all treatment groups exhibited increase in body weight throughout the four weeks of treatment. Rats from NC group showed a steady pattern of body weight gain while DC group appeared to gradually lose body weight after STZ administration. As shown in Table 1, at the end of treatment period, rats treated with MET and both MO-SeNPs dosage showed noticeable increase from DC group with 0.25 mg/kg MO-SeNPs treatment showed statistically significant changes.

**Table 1.** Comparison table for the effect of different treatments towards diabetic group, diabetic and normal control on the FBG level, insulin, body weight, HOMA-IR and HOMA-β% after four weeks of treatment.

Groups	Final FBG (mmol/L)	Final Insulin (μIU/mL)	Final Body Weight (g)	HOMA-IR	HOMA-β%
NC	5.45 ± 0.264 ^b^	9.6 ± 0.954 ^b^	272 ± 15.7 ^b^	2.35 ± 0.23 ^b^	95.75 ± 9.52 ^b^
DC	26.3 ± 1.47 ^a^	16.4 ± 1.05 ^a^	169 ± 19.7 ^a^	18.33 ± 1.18 ^a^	15.05 ± 0.97 ^a^
PC	19.6 ± 0.951 ^a^	8.37 ± 1.75 ^b^	235 ± 12.6	7.08 ± 1.48 ^b^	12.25 ± 2.56 ^a^
Tx 0.25	7.77 ± 1.59 ^b^	5.72 ± 0.955 ^b^	251 ± 20.1 ^b^	2.27 ± 0.38 ^b^	21.05 ± 3.51 ^a^
Tx 0.5	17.2 ± 4.15 ^a,b^	10.3 ± 2.41	217 ± 14.2	7.86 ± 1.84 ^a,b^	15.08 ± 3.53 ^a^

The values are expressed as mean + SEM (n=6). Significant value *(p<0.05)* are noted as letters (a) and (b) which expressed significant different to NC and DC respectively.

### Fasting blood glucose level

**Figure 3.**
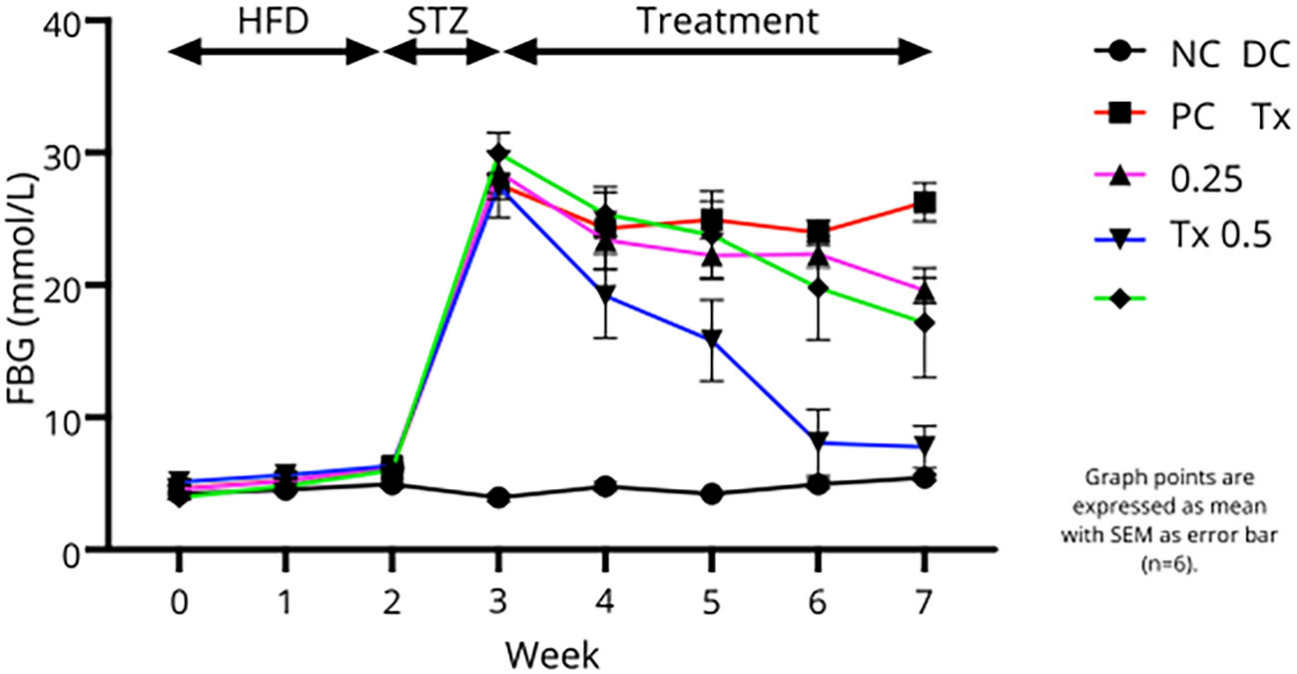
Pattern of FBG level of the animals groups throughout the experimental period. Graph points are expressed as mean with SEM as error bar (n=6).

Based on Figure. 3, animal groups that received HFD showed a slightly higher FBG throughout the first two week compared to the NC group. STZ injection to the diabetic groups post-HFD showed a remarkable increase in FBG level, passing the benchmark for confirmation of diabetes (16.7 mmol/L). During four weeks of treatment, rats treated with metformin, 0.25 mg/kg and 0.5 mg/kg MO-SeNPs showed a decreasing trend of FBG level but with different efficiency. Interestingly, based on Table 1, both MO-SeNPs treated groups showed significant reduction of FBG level after four weeks of treatment compared to DC group with Tx 0.25 group values nearly reaching the normal rats.

### Serum insulin, HOMA-IR and HOMA-β% profile

In this experiment, DC group showed the highest insulin level, HOMA-IR index and low values of HOMA-β%. Table 1 demonstrated that rats that receive both dosage treatment of MO-SeNPs and MET after four weeks, showed much lower serum insulin level as compared to DC rats and with MET and 0.25 mg/kg MO-SeNPs administration exhibit statistically significant different of the insulin values. Furthermore, HOMA-IR index showed that both MO-SeNPs dosage and MET treatment produced a significant reduced from DC values with lower dose of MO-SeNPs (0.25 mg/kg) showed lower index than NC group. However, the β-cell function from HOMA-β% values showed that each treatment group did not reach a significant improvement in β-cell function comparing to DC rats.

### Serum liver and lipid biochemical profiles

Based on Table 2, induction of diabetes by HFD/STZ showed to cause elevated level of serum hepatic enzyme like ALT, AST, ALP which reflected by DC group through the experiment. Moreover, it also showed a highest value for serum lipid biomarkers such as LDL, TGL and cholesterol with lowest value of HDL among other experimental groups. After 4 weeks of treatment period, MO-SeNPs and MET administration have shown to alleviate the values for the serum liver and lipid profiles. Tx 0.25 group have shown to exhibit significant changes on the level of ALT, AST and ALP level while Tx 0.5 group showed significant improvement on AST, HDL and LDL level as compared to DC group. Effect of metformin treatment exhibited a significant change towards TGL level as compared to diabetic control rats. However, cholesterol had shown no noteworthy differences among every treatment groups.

**Table 2.** Biochemical profile assessment results towards experimental groups on the ALT, AST, ALP, HDL, LDL, TGL and cholesterol level after four weeks of treatment.

Groups	ALT (U/L)	AST (U/L)	ALP (U/L)	HDL (mmol/L)	LDL (mmol/L)	TGL (mmol/L)	Cholesterol (mmol/L)
NC	55.17 ± 3.591 ^b^	168.3 ± 16.67 ^b^	269.3 ± 23.02 ^b^	1.04 ± 0.208 ^b^	0.137 ± 0.019 ^b^	0.928 ± 0.061 ^b^	1.152 ± 0.107
DC	171.5 ± 39.5 ^a^	352.7 ± 89.45 ^a^	1342 ± 339.3 ^a^	0.525 ± 0.015 ^a^	0.327 ± 0.053 ^a^	1.737 ± 0.095 ^a^	1.353 ± 0.083
PC	118 ± 39.23	179.5 ± 20.7	1272 ± 211 ^a^	0.793 ± 0.092	0.263 ± 0.024	0.963 ± 0.335 ^b^	1.328 ± 0.121
Tx 0.25	66.33 ± 8.468 ^b^	137.3 ± 7.451 ^b^	533.7 ± 111.6 ^b^	0.747 ± 0.036	0.288 ± 0.027 ^a^	1.075 ± 0.179	1.323 ± 0.044
Tx 0.5	114 ± 22.97	147.5 ± 18.31 ^b^	1284 ± 90.43 ^a^	1.05 ± 0.095 ^b^	0.168 ± 0.036 ^b^	1.102 ± 0.112	1.238 ± 0.048

The values are expressed as mean + SEM (n=6). Significant value *(p<0.05)* are noted as letters (a) and (b) which expressed significant different to NC and DC respectively.

### Serum antioxidant enzyme activity and hepatic lipid peroxidation analysis

**Figure 4.**
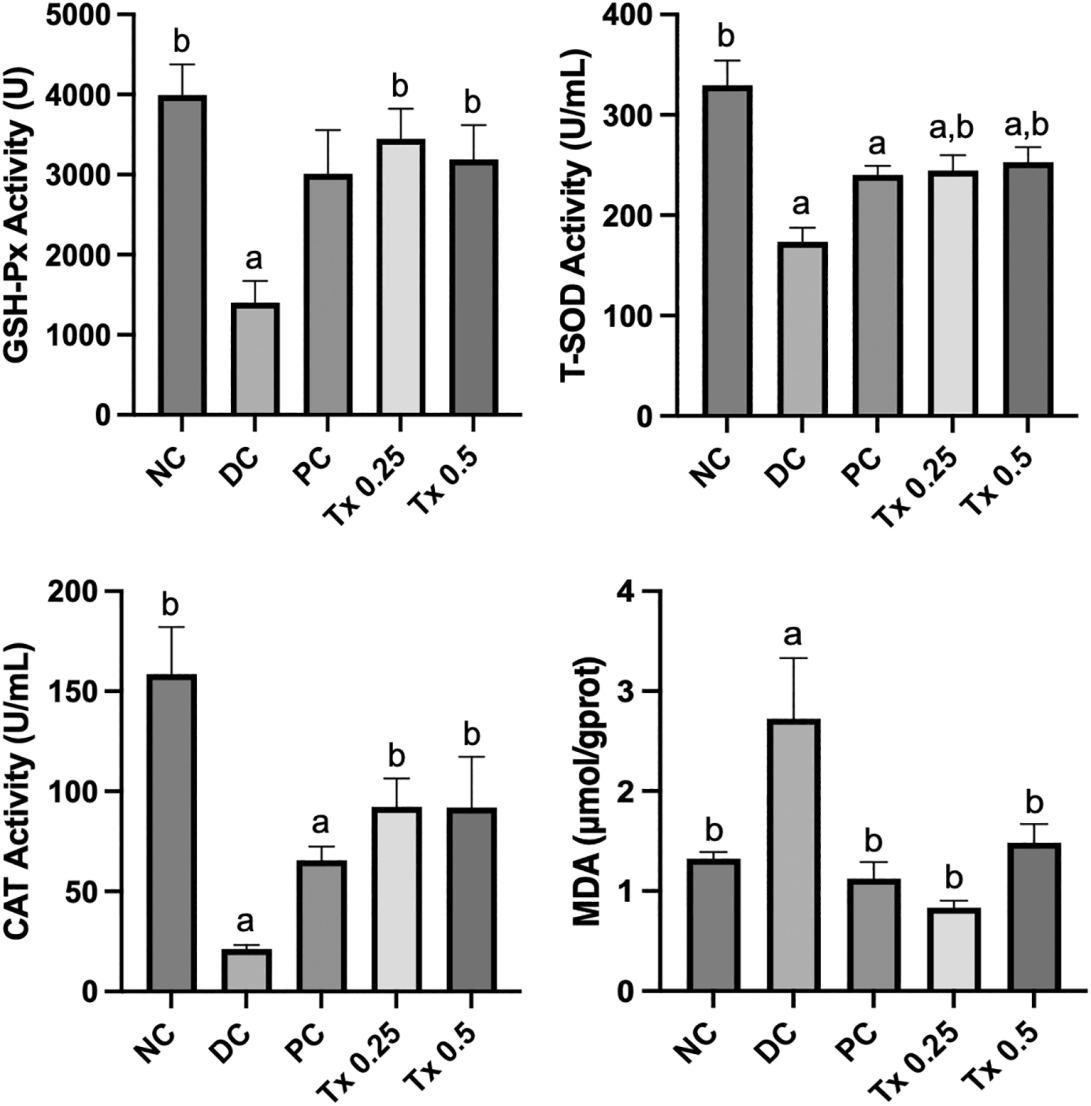
Visualisation of MO-SeNPs' effects on enzymatic antioxidant activity and lipid peroxidation after four weeks. The graphs are expressed as mean + SEM (n=6). Significant value *(p<0.05)* is noted as letters (a) and (b) which expressed significant different to NC and DC respectively.

In order to explore the capacity of MO-SeNPs treatment on its ability to defend against oxidative damage, the level of serum enzymatic antioxidant was assessed in this research. Referring to Figure. 4, throughout the four weeks of treatment with MO-SeNPs, the treated rats showed a significant alleviation all enzymatic antioxidant such as GSH-Px, CAT, T-SOD as compared to the diabetic control rats. Moreover, both MO-SeNPs dosage did not showed a staggering difference of values when being compared with each other with 0.25 mg/kg MO-SeNPs to be slightly higher at GSH-Px (3449 ± 371.6 U) and CAT (92.32 ±14.09 U/mL) while 0.5 mg/kg MO-SeNPs being vaguely higher at T-SOD activity level (252.8 ± 14.98 U/mL). Meanwhile, MDA level for hepatic lipid peroxidation assessment proved that both dosage of MO-SeNPs treatments to be significantly effective for the protection against oxidative damage towards the level with 0.25 mg/kg MO-SeNPs to be most effective with values much lower than NC group. Our results also exhibited that DC groups possess lowest enzymatic antioxidant activity and highest impact on lipid peroxidation.

### Hepatic inflammatory cytokine level analysis

**Figure 5.**
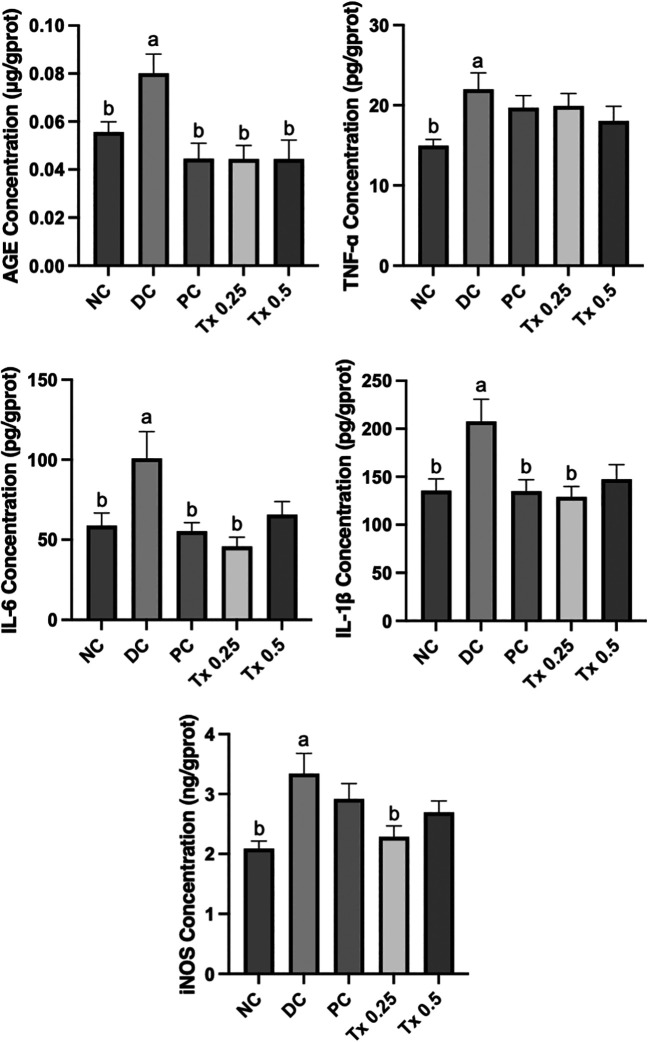
The effect of inflammatory cytokine level of liver tissue after four weeks of daily MO-SeNPs treatments. The graphs are expressed as mean + SEM (n=6). Significant value *(p<0.05)* is noted as letters (a) and (b) which expressed significant different to NC and DC respectively.

To study the efficacy of MO-SeNPs influence towards cytokine that are pro-inflammation, assessment towards biomarkers level such as AGE, TNF-α, IL-6, IL-1β and iNOS were done on the liver tissue of experimental rats. From Figure 5, the untreated DC group showed the highest level of inflammatory cytokines tested in this study. On the other hand, experimental groups that received both MO-SeNPs and MET have shown improvements and decreases in values. Similar to antioxidant activity status, the lower dosage of MO-SeNPs (0.25 mg/kg) exhibited much significant changes towards hepatic AGE, IL-6, IL-1β and iNOS as compared to DC group while another MO-SeNPs treatment (0.5 mg/kg) managed to cause significant improvement on hepatic AGE level. However, both dosage treatments of MO-SeNPs and MET did not show a significance improvement from TNF-α despite having a lower value as compared to diabetic control rats.

## Discussion

In this study, we utilized the combination of high fat diet and streptozotocin (STZ) to induce type 2 diabetes mellitus (T2DM). This model principal lies on the early predisposition of obesity symptoms followed injection of STZ injection which highly reported to be toxic towards β-cell at the pancreas, causing cell death and reduction of β-cell mass. ^24^ Besides, the administration of HFD/STZ in animal models exhibited T2DM characteristics such as hyperglycemia, hyperinsulinemia, and dyslipidaemia. ^25^ Based on our results, the diabetic control group showed higher water and food intake compared to other groups, which suggest symptoms of polyphagia and polydipsia. Metabolic disorder like T2DM is closely associated with insulin resistance, dysregulation of metabolic process and hormonal imbalance, impeding with nutrients metabolism for the body and cause uncontrolled brain signalling for hunger. ^26, 27^ Besides that, the body response trying to compensate for hyperglycaemic condition, causing increased water depletion by excretion (polyuria), and ultimately activating thirst centre to recover water loss. ^28^ Despite an increase in food and water intake in this study, our results showed a decreasing pattern of body weight towards diabetic rats after administration of STZ. The weight gain observed following two weeks of high-fat diet (HFD) begins to decline after the administration of STZ injection. The STZ accelerates the onset of diabetes, leading to weight loss due to the catabolism of muscle and adipose tissues, which is associated with the body’s inability to utilize glucose for energy. ^29^ Furthermore, our DC rats showed persistent elevation of FBG and insulin level in the serum which were classic features of T2DM. Conversely, diabetic rats treated with both doses of MO-SeNPs showed a reversal of all these symptoms. This effect might be attributed to selenium’s insulin mimetic characteristic, which help to restore β-cell activity, stimulate insulin release, and lower blood glucose levels. ^30^ In this study, the value of HOMA-IR shown to be consistent with FBG and insulin level among the groups treated with MO-SeNPs which significantly lower than DC group. This may recommend that MO-SeNPs managed to improved sensitivity towards insulin and stimulate cell glucose uptake, hence reducing serum insulin and FBG level. ^31^ However, β-cell percentage index from our results showed no significant improvement as compared to DC group which could be due to insufficient treatment duration. Previous reports have shown that prolong SeNPs treatment period up to 6 and 8 weeks may help to recover higher β-cell mass at the pancreas. ^25, 32^

The liver played a crucial role for blood glucose homeostasis, metabolic functions, storage, detoxification, and excretion. ^33^ T2DM are commonly associated with non-alcoholic fatty liver disease (NAFLD) which can be assessed by measuring the level of liver enzymes biomarkers like ALT, AST and ALP. ^34^ Increased serum activities of transaminases, ALP in T2DM-induced rats can happen from STZ-induced hepatic injury, leading to enzyme leakage from the liver tissue into the blood. ^35^ Based on our results, the liver enzyme profile analysis showed significant improvements in ALT, AST and ALP levels in the Tx 0.25 treatment groups, which recommends a hepatoprotective effect of the lower dosage of MO-SeNPs. While 0.5 mg/kg MO-SeNPs did not provide statistically significant improvement towards ALT and ALP, the general patterns point to the ability of MO-SeNPs in ameliorating diabetic liver damage.

Multiple reports have shown that T2DM often involves aberrations in serum lipid profiles (dyslipidemia) which were characterised by elevated TG, LDL, cholesterol and decreased HDL levels. ^36, 37^ In this study, diabetic rats showed significant disorders in their serum lipid profiles. Both dosages of MO-SeNPs led to improvements in HDL and LDL levels; however, only the higher dosage of MO-SeNPs (0.5 mg/kg) resulted in significant changes. This may suggest MO-SeNPs pronounced effect on lipoproteins to be dosage-dependent. Previous study also reported that HDL level to be directly proportional to the amount of SeNPs supplementation. ^38^ Increased HDL level also encourages efflux of cholesterol and reduced the risk for development of atherosclerotic plaque which may lead to cardiovascular problem. ^39^ Efflux of cholesterol from liver may also upregulate the expression of LDL receptor, mediated the uptake of LDL, hence reducing serum LDL level. However, due to lack of significant improvement in triglyceride levels across both SeNPs dosages, it may require a longer treatment period to achieve better results. Besides that, only metformin treatment showed significant improvement on TGL level. It has been reported that metformin induces triglyceride lipolysis through hormone-sensitive lipase and AMP-activated protein kinase stimulation. ^40^

Multiple reports have highlighted on the action of oxidative stress from ROS accumulation as the main factor for T2DM progression, causing complications in humans and animal, such as nonspecific oxidative injury to DNA, proteins, molecules, ultimately leads to alteration of their configuration and disrupts their function. ^41^ Extreme high blood glucose environment in T2DM generally influence the elevation of oxidative stress in tissues and cause harmful unbalance between ROS production and the antioxidant defense activity like GPx and SOD. ^42^ Based on our result, diabetic control group marked the lowest antioxidant activity level which represent the impaired antioxidant function in HFD/STZ-induced rats. The antioxidant enzyme activity observed in this study further highlights the protective effects of MO-SeNPs. The significant increases in GSH-Px, CAT, and T-SOD activities in both of MO-SeNPs treated groups signifies that each dosage effectively boost the antioxidant defense system. The improvement of antioxidant enzyme level may be contributed by MO-SeNPs characteristic to primarily attributed to increasing GPx enzyme expression, recuperation of hyperglycaemic condition and its unique nanosized dimension which allows large surface area and better bioavailability. ^25, 43^ Moreover, the hepatic MDA level observed in this study further highlights the protective effects of MO-SeNPs. MDA is a biomarker that was produced as the end product of lipid peroxidation, showed a significant increase in level in DC group. ^44^ MO-SeNPs treatment remarkably reduced hepatic MDA levels, suggesting a significant declined in lipid peroxidation activity in liver tissue.

In this study, the impact of MO-SeNPs on inflammatory cytokines associated with diabetes were also investigated. Hyperglycaemic condition can trigger damage towards tissues and organs through mechanisms involving AGEs formation and pro-inflammatory cytokines in liver tissues. ^45^ Besides that, previous studies on human and animal models show a direct association between inflammation and insulin resistance in T2DM development. ^46^ Based on our results, HFD/STZ induced rats showed significant rise in hepatic AGEs, TNF-α, IL-6, IL-1β, and iNOS levels, which were stabilized by the administration of MO-SeNPs. Despite notable decrease in the inflammatory cytokines level, TNF-α showed no significant changes against both MO-SeNPs dosages. This result may suggest that while MO-SeNPs are efficient in regulating the inflammatory reaction, they might be selective and cannot completely suppress all pro-inflammatory pathways at the given doses. The lower MO-SeNPs dose (0.25 mg/kg) demonstrated the best results in restoring pro-inflammatory cytokine production in hepatic tissues, which recommends that it might be the optimal dosage for targeted biological efficacy with maybe lower adverse effects.

## Conclusions

In a nutshell, MO-SeNPs exhibited a significant hypoglycemic, antioxidative and anti-inflammatory properties towards T2DM rat model with the lower dosage to be more effective. This therapeutic effect contributed to reducing oxidative stress and inflammatory damage towards the liver, restoring the insulin sensitivity and reducing lipid peroxidation, hence diminishing the risk for complication of diabetes. However, further dosage determination and toxicity study are needed to elucidate more on the pharmacokinetics action of MO-SeNPs.

## Ethical approval

Ethical approval for animal experiment from MSU Ethic Committee (EA-L3-01-SGS-2024-03-0002) on 23 ^rd^ March 2024. This article is reported in line with the ARRIVE (Animal Research: Reporting of in vivo Experiments) guidelines. ^48^

## Data Availability

Figshare: Raw underlying data for the Antioxidant and hepatoprotective effects of Moringa oleifera-mediated selenium nanoparticles in diabetic rat. https://doi.org/10.6084/m9.figshare.27859794.v2. ^47^ The project contains the following underlying data:
• Raw Underlying Data Data are available under the terms of the Creative Commons Attribution 4.0 International license (CC-BY 4.0). Figshare: ARRIVE guideline checklist for ‘Antioxidant and hepatoprotective effects of Moringa oleifera-mediated selenium nanoparticles in diabetic rat https://doi.org/10.6084/m9.figshare.27936879.v1. ^48^ The project contains the following reporting guidelines:
• The-ARRIVE-Author-Checklist-Full.pdf Data are available under the terms of the Creative Commons Attribution 4.0 International license (CC-BY 4.0).
