# Supplementary material for: Antioxidant and Hepatoprotective Effects of Moringa oleifera-mediated Selenium Nanoparticles in Diabetic Rats
Source: F1000Res. 2025 Jan 2;14:7. [Version 1] doi: 10.12688/f1000research.159362.1 (PMC12515360; doi:10.12688/f1000research.159362.1)
Supplement: Peer review history

# Reviewer report: Reviewer response for version 1

**DOI:** 10.5256/f1000research.175079.r418287

**Published:** 2025-10-22

**Authors:** Mahmoud Balbaa

**Affiliations:** 1 Department of Biochemistry, Faculty of Science, Alexandria University, Alexandria, Egypt

There are problems in the units of some parameters:

 1) In Figure 4, Are the antioxidant enzymes and MDA measured in serum? If so, the unit of MDA should be expressed per ml. If these parameters were measured in this issue, their units have to be expressed per mg of protein (mg protein).

 2) The data reported in Figure 5 should be provided as levels rather than concentrations, and this level should be expressed per milligram rather than gram of protein.

Is the work clearly and accurately presented and does it cite the current literature?

Partly

If applicable, is the statistical analysis and its interpretation appropriate?

Yes

Are all the source data underlying the results available to ensure full reproducibility?

Partly

Is the study design appropriate and is the work technically sound?

Yes

Are the conclusions drawn adequately supported by the results?

Yes

Are sufficient details of methods and analysis provided to allow replication by others?

Yes

Reviewer Expertise:

Cell signaling, enzymes in health and disease, and growth factors.

I confirm that I have read this submission and believe that I have an appropriate level of expertise to confirm that it is of an acceptable scientific standard, however I have significant reservations, as outlined above.

# Reviewer report: Reviewer response for version 1

**DOI:** 10.5256/f1000research.175079.r418286

**Published:** 2025-10-11

**Authors:** Shobana Chandrasekar

**Affiliations:** 1 Vels Institute of Science, Technology and Advanced Studies (VISTAS), Chennai, India

I found the paper and prepared a full, structured report: concise summary, expanded methodological details, results with the main data points the authors report, interpretation (mechanisms), strengths & limitations, and suggested next steps (experiments and translational considerations). The study presents convincing preclinical evidence that *Moringa oleifera*-mediated selenium nanoparticles improve glycaemic control, boost hepatic antioxidant defenses, reduce inflammation and protect liver tissue in a rat model of T2DM.

Is the work clearly and accurately presented and does it cite the current literature?

Yes

If applicable, is the statistical analysis and its interpretation appropriate?

Yes

Are all the source data underlying the results available to ensure full reproducibility?

Yes

Is the study design appropriate and is the work technically sound?

Yes

Are the conclusions drawn adequately supported by the results?

Yes

Are sufficient details of methods and analysis provided to allow replication by others?

Yes

Reviewer Expertise:

Neuroscience, Diabetes, Community Nutrition

I confirm that I have read this submission and believe that I have an appropriate level of expertise to confirm that it is of an acceptable scientific standard.

# Reviewer report: Reviewer response for version 1

**DOI:** 10.5256/f1000research.175079.r418285

**Published:** 2025-09-30

**Authors:** Neihaya Heikmat Zaki

**Affiliations:** 1 Department of Biology, College of Science, Mustansiriyah University, Baghdad, Iraq

Biosynthesized SeNPs treatment significantly reduced blood glucose levels and restored insulin resistance, with a lower dose demonstrating better glycemic control than a larger dose. MO-SeNPs also increased hepatic antioxidant enzyme activity.

 The work is clear, and the study design is appropriate. Also, the methods are sufficient. The statistical analysis is appropriate too. Data in the results are also reproducible.

Is the work clearly and accurately presented and does it cite the current literature?

Yes

If applicable, is the statistical analysis and its interpretation appropriate?

Yes

Are all the source data underlying the results available to ensure full reproducibility?

Yes

Is the study design appropriate and is the work technically sound?

Yes

Are the conclusions drawn adequately supported by the results?

Yes

Are sufficient details of methods and analysis provided to allow replication by others?

Yes

Reviewer Expertise:

Biotechnology, Nanotechnology and microbiology.

I confirm that I have read this submission and believe that I have an appropriate level of expertise to confirm that it is of an acceptable scientific standard.

# Reviewer report: Reviewer response for version 1

**DOI:** 10.5256/f1000research.175079.r390429

**Published:** 2025-06-23

**Authors:** Wira Eka Putra

**Affiliations:** 1 Universitas Negeri Malang, Jawa Timur, Indonesia

The manuscript entitled "Antioxidant and Hepatoprotective Effects of Moringa oleifera-mediated Selenium Nanoparticles in Diabetic Rats" is clearly written and presents interesting findings. However, several important points require clarification and revision:

1.  Please include a schematic diagram or a table summarizing the experimental groups, treatment regimens, and duration. This will help readers better understand the study design.
2. Are there any statistically significant differences in body weight after treatment (Figure 1)? Please indicate the statistical analysis results in the figure or legend.
3. Please clarify whether the observed reduction in FBG levels after treatment is statistically significant (Figure 3). If not, this should be discussed in the context of the findings.
4.  Several bars lack statistical annotation (Figure 4 and 5). Please ensure all groups are consistently annotated to reflect statistical significance.
5. Kindly include electron microscopy images of the synthesized MO-SeNPs to validate their morphology and size distribution.
6. Since STZ induces β-cell toxicity and the discussion mentions the potential of selenium to restore β-cell function, it would strengthen the manuscript to include histopathological images of the pancreas to support this claim.
7. Based on the presented results, could the authors suggest a possible mechanism of action? This could be supported by referencing prior evidence or public databases. Alternatively, I recommend conducting additional experiments to explore the underlying molecular pathways involved.

Is the work clearly and accurately presented and does it cite the current literature?

Yes

If applicable, is the statistical analysis and its interpretation appropriate?

Yes

Are all the source data underlying the results available to ensure full reproducibility?

Partly

Is the study design appropriate and is the work technically sound?

Yes

Are the conclusions drawn adequately supported by the results?

Yes

Are sufficient details of methods and analysis provided to allow replication by others?

Partly

Reviewer Expertise:

Immunology

I confirm that I have read this submission and believe that I have an appropriate level of expertise to confirm that it is of an acceptable scientific standard, however I have significant reservations, as outlined above.
